# Supplementary material for: Bone Infarcts and Tumorigenesis—Is There a Connection? A Mini-Mapping Review
Source: Int J Environ Res Public Health. 2022 Jul 29;19(15):9282. doi: 10.3390/ijerph19159282 (PMC9367991; doi:10.3390/ijerph19159282)
Supplement: Supplementary file 1 [file ijerph-19-09282-s001.zip › ijerph-1811655-supplementary.pdf]

Supplementary file S1. Search phrases.

Google Scholar search phrases:

- ("bone infarct") AND (cancer OR radiotherapy OR leukemia OR neoplasm* OR carcinogenesis OR tumorigenesis OR sarcoma) – 515 results

Robbin Mc Donald, Spazzioli, Alhamnnjbj, Lewin, Sieben Cahraman Cenitas

- ("AVN" OR "avascular necrosis") AND (cancer OR radiotherapy OR leukemia OR neoplasm* OR carcinogenesis OR tumorigenesis OR sarcoma) – 14 100 results

Total: 14615.
Cochrane database:

Search Name:
Date Run: 21/07/2022 21:21:11
Comment:

ID	Search	Hits
#1	("avascular necrosis"):ti,ab,kw (Word variations have been searched)	397
#2	("AVN"):ti,ab,kw (Word variations have been searched)	83
#3	("bone infarct"):ti,ab,kw (Word variations have been searched)	6
#4	(leukemia):ti,ab,kw (Word variations have been searched)	15446
#5	("neoplasma"):ti,ab,kw (Word variations have been searched)	13
#6	("Cancer"):ti,ab,kw (Word variations have been searched)	182802
#7	(carcinogenesis):ti,ab,kw (Word variations have been searched)	1162
#8	(tumorigenesis):ti,ab,kw (Word variations have been searched)	239
#9	(sarcoma):ti,ab,kw (Word variations have been searched)	2690
#10	MeSH descriptor: [Osteonecrosis] explode all trees	292
#11	#1 OR #2 OR #3	441
#12	#4 OR #5 OR #6 OR #7 OR #8 #9	192433
#13	#11 AND #12 with Cochrane Library publication date Between Jan 2012 and Jul 2022	26
#14	#13 OR #10 with Cochrane Library publication date Between Jan 2012 and Jul 2022	212 – results

TOTAL: 212 results
